# Navigating the factor zoo around the world: an institutional investor perspective

**DOI:** 10.1007/s11573-021-01035-y

**Published:** 2021-04-26

**Authors:** Söhnke M. Bartram, Harald Lohre, Peter F. Pope, Ananthalakshmi Ranganathan

**Affiliations:** 1 Centre for Economic Policy Research (CEPR), London, UK; 2 Warwick Business School, Finance Group, University of Warwick, Coventry, CV4 7AL UK; 3 Invesco Quantitative Strategies, An der Welle 5, 60322 Frankfurt am Main, Germany; 4 Centre for Endowment Asset Management (CEAM) at Cambridge Judge Business School, University of Cambridge, Cambridge, UK; 5 Centre for Financial Econometrics, Asset Markets and Macroeconomic Policy (EMP), Lancaster University Management School, Bailrigg, Lancaster, LA1 4YX UK; 6 Department of Accounting, Bocconi University, Via Roentgen 1, 20136 Milano, Italy

**Keywords:** Asset pricing, Factor investing, Mispricing, Risk factor, Institutional investor, Transaction costs, Limits to arbitrage, Market efficiency, Anomaly, G11, G14, G15

## Abstract

The literature on cross-sectional stock return predictability has documented over 450 factors. We take the perspective of an institutional investor and navigate this zoo of factors by focusing on the evidence relevant to the practicalities of factor-based investment strategies. Establishing a sound theoretical rationale is key to identifying “true” factors, and we emphasize the need to recognize data-mining concerns that may cast doubt on the relevance of many factors. From a practical investment perspective, much of the factor evidence documented by academics may be more apparent than real. The performance of many factors is dependent on the inclusion of small- and micro-cap stocks in academic studies, although such stocks would likely be excluded from the real investment universe due to illiquidity and transaction costs. Nevertheless, a parsimonious set of factors emerges in equities and other asset classes, including currencies, fixed income, and commodities. These factors can serve as meaningful ingredients to factor-based portfolio construction.

## Introduction

Equity portfolios tilted towards observed firm characteristics, or factors, have attracted considerable attention from scholars and investment practitioners. From an academic perspective, characteristic-based factors are often used to explain the cross-section of equity returns, with a parsimonious subset for priced factors in modeling equity risk. From an investment perspective, the objective is to harness associated return premia when constructing factor-based equity portfolios. Whether such premia exist as compensation for bearing undiversifiable risk or as reward for identifying mispricing, they are seen as the holy grail of factor investing strategies. Against this backdrop, it is not surprising that the factor literature has proliferated to what is now considered a “zoo of factors” (Cochrane 2011), containing more than 450 predictive factors.

**Fig. 1:**
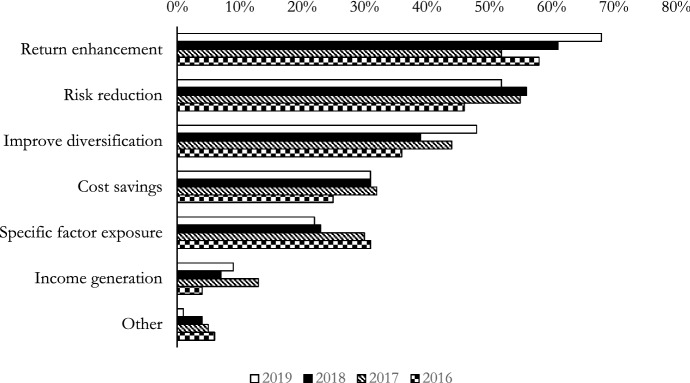
Investment objectives of institutional investors. The figure shows the most common objectives of asset owners when evaluating factor investing strategies. It compares the changes in priority of objectives of the survey participants from 2016–2019. Source: 2019 FTSE Smart Beta Global Survey

The factor zoo’s inhabitants are diverse. To illustrate, value factors combine information from financial statements and market prices to identify relatively cheap stocks, while momentum and reversal factors are constructed from past return series. Quality factors build on accounting numbers to identify firms with strong balance sheets and lower downside risk, while low volatility strategies exploit the covariance structure of stock returns to establish defensive portfolio strategies that generate higher risk-adjusted returns. As they embody different styles of investing, factor-based strategies promise tailored exposures to meet risk-return objectives at lower costs, appealing to institutional investors who seek to improve diversification and control specific risk factor exposures (Fig. 1). This can also be seen from the 2019 FTSE Smart Beta Global Survey, which expects the adoption of such factor strategies by institutional investors to grow, especially those marketing exchange-traded funds. Furthermore, the survey reports that an increasing number of institutional investors plan to adopt a factor lens in search of parsimonious and holistic approaches to asset management.

Factor investing appeals to investors as it is built on solid theoretical and empirical foundations, with a rationale for why factors worked in the past and are expected to continue to work in the future. Persistent factor performance is likely if a factor captures undiversifiable, systematic risks for which investors demand compensation. However, persistent investor biases are often also invoked as plausibly contributing to systematic mispricing of securities. In the absence of systematic biases, mispricing should be transient, and the associated return predictability should be short-lived, unless the underlying biases continue to exist and there are reasons to believe that mispricing cannot be arbitraged away.

While early factor research focuses on establishing and rationalizing single factors, recent literature features several important studies that replicate many published factors to analyze the cross-section of predictors (e.g. Green et al. 2017; Hou et al. 2020b; Feng et al. 2020). Replication studies are key in validating proposed factors, ideally confirming factor evidence on the same data and sample period as well as providing corroborating evidence for other time periods and samples, see Jensen et al. (2021). Moreover, Harvey et al. (2016) use statistical techniques to account for data snooping biases to separate true factors from false in a set of 316 published factors. They also describe how certain factors were deemed “significant” by luck. Pukthuanthong et al. (2019) use principal component analysis to test whether a given systematic risk factor qualifies as genuine. By explaining the need to distinguish priced factors from predictor characteristics, they propose several innovative methods for evaluating factors. Such guidelines are important from an investor perspective to avoid disappointing performance of their factor portfolios.

The majority of factors documented in the literature has first been identified in the US equity market. Subsequently, the predictive ability of some factors has also been replicated in international markets—including developed, emerging and even frontier markets—as well as in other asset classes. Such evidence can be viewed as out-of-sample evidence, despite meaningful differences across countries and asset classes with regard to institutional features such as transaction costs, liquidity, factor crowding, and the number of investible assets. Of course, exceptions exist, such as the weaker performance of momentum factors in Japan compared with other markets.

The empirical evidence, especially in early studies, often focuses on returns of equally weighted factor portfolios, which may overstate the realizable factor returns if less investible small- and micro-cap stocks are important to factor performance. Transaction costs are higher for difficult-to-arbitrage stocks, such as microcaps, low liquidity, and high idiosyncratic volatility stocks. In a related vein, (factor) investors may face short-selling constraints, which may limit the potential factor performance to the contribution of a factor’s long leg. Also, the portfolio turnover implied by a strategy is an important determinant of realizable factor performance—a low-turnover value strategy will incur significantly lower transaction costs than higher-turnover factors, such as momentum or short-term reversal. Textual analysis and the application of machine learning techniques are among recent developments in factor research, for instance to develop new or identify a robust set of factors. Finally, broad factor concepts such as carry, value, momentum, and quality apply in many asset classes, suggesting to approach factor investing through a multi-asset multi-factor lens.

From an investment perspective, there are several key aspects for investors to consider when adopting factors in the investment process. First, despite hundreds of factors proposed in the literature, the number of factors that contain independent and exploitable predictive information for the cross-section of asset returns is much smaller. Second, with the increasing availability and growth in computational power facilitating the exploitation of alternative data sources, controlling data snooping biases is key to avoiding false discoveries. Third, the evidence on factor performance is often sensitive to the selected investment universe, with returns depending on the ability to invest in small and micro-cap stocks. Such factors are irrelevant for institutional investors, because the amount of capital that can be deployed is limited, and because market impact and other transaction costs make it expensive to trade in such stocks. Accounting for such real-world frictions is important, and investors should focus on whether a given factor delivers significant performance in value-weighted portfolios after accounting for transaction costs and investment constraints related to institutional investors’ mandates.

## Notable species in the factor zoo

### A bird’s eye perspective

**Fig. 2:**
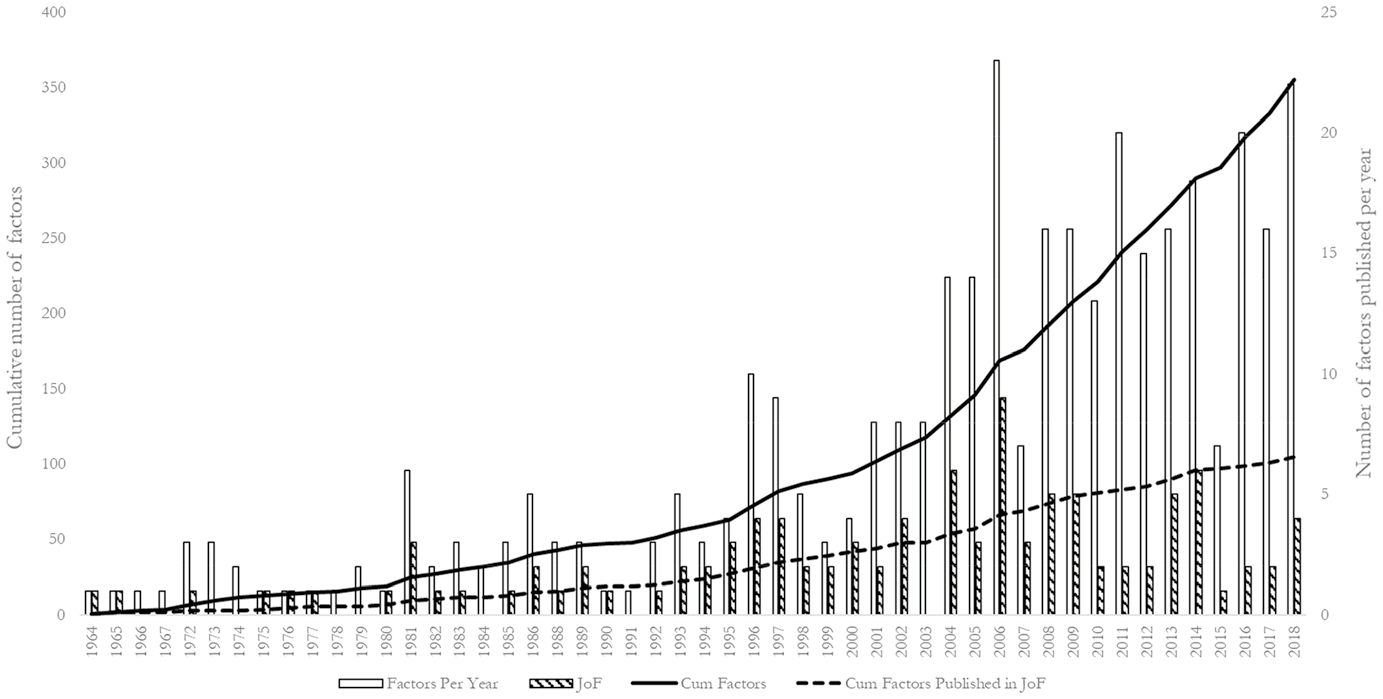
Proliferation of factors since 1964. The figure shows the trend in the year-wise publication of factor studies in well-known finance journals and the number of factor studies published in the top three finance journals: Journal of Finance, Journal of Financial Economics, and Review of Financial Studies since 1964. Source: Factor Census dataset of Harvey and Liu (2019)

Starting with Cochrane (2011), academics have attempted to address concerns about the expanding factor zoo. Harvey and Liu (2019) conduct a factor census to manage the growing number of factors. Figure 2 shows the cumulative growth in the number of published factors in the top three finance journals since 1964, with 105 papers published exclusively in the *Journal of Finance*. Since 2008, there has been nearly exponential growth in the number of published articles, and hence in what has become known as the factor zoo. Supporting this view, a recent publication by Hou et al. (2020b) documents 452 factors that researchers have uncovered.

A factor is typically based on an asset characteristic (or predictor variable) that has power for explaining the cross-section of future asset returns. If the ensuing factor premium is found to compensate for risk, it is considered a risk factor. Conversely, if the factor premium is not predicted to capture risk by theory and cannot be rationalized with generally accepted asset pricing models, it is considered an anomaly or a mispricing factor. However, there is often ambiguity in the literature with respect to assigning a given predictive factor to either category, risk or mispricing, partly because theoretical understanding evolves inductively and dynamically as empirical regularities are uncovered.

Researchers have suggested several guidelines to identify ‘true’ factors (those that generate persistent expected returns as a result of bearing priced risk or exploiting persistent behavioral biases or structural impediments). True factors should have incremental explanatory power over previously identified factors (Feng et al. 2020). The returns to true factors are persistent over time, pervasive across samples (e.g. countries, asset classes), and can withstand definitional variations. To be implementable, any given factor needs to survive transaction costs and have a solid theoretical rationale for the existence of the associated premia. However, time variation may make it challenging to distinguish empirically between factor premia and mispricing. Before we look into how to delineate risk and mispricing, we will first introduce the more traditional style factors and corresponding asset pricing models that are typically used to rationalize new predictive factors.

### Salient factors and asset pricing factor models

Starting with the capital asset pricing model (CAPM), which introduced the relationship between average returns and market exposure (or market beta), researchers have been keen to identify a model that best explains the cross-section of asset returns (see Table 1). Such models are of interest to academics and practitioners alike, as they are expected to help detect robust patterns in asset returns, which can be used to formulate profitable investment strategies and control portfolio risk. However, the empirical evidence challenges the CAPM. For instance, the low volatility factor is a rebuttal to the CAPM as seen from Haugen and Heins (1972), who find that low-risk stocks yield higher risk-adjusted returns than high-risk stocks over the long run.

**Table 1:** Prominent asset pricing models

Model	Study	Factor(s)	MS_q_	MS_q5_	GRS_72_	GRS_34_
(1)	(2)	(3)	(4)	(5)	(6)	(7)
CAPM	Sharpe (1964); Lintner (1965); Mossin (1966)	Market	0.1			3.95***
Fama and French 3-factor model	Fama and French (1993)	Market, size, and value	0.21		2.10***	3.70***
Carhart 4-factor model	Carhart (1997)	Market, size, value and momentum	0.3			3.10***
Fama and French 5-factor model	Fama and French (2015)	Market, size, value, profitability, and investment		0.32	1.79***	2.60***
Fama and French 4-factor model	Fama and French (2015)	Market, size, profitability, and investment				
*q-*factor model	Hou et al. (2015)	Market, size, investment, and profitability	0.43	0.42		2.42***
Stambaugh and Yuan 4-factor model	Stambaugh and Yuan (2017)	Market, size, management and performance		0.41	1.54***	1.71***
*q5-* model	Hou et al. (2018)	Market, size, investment, profitability and expected investment growth		0.63	1.78***	
Fama and French 5-factor model plus momentum	Fama and French (2018)	Market, size, value, profitability, investment and momentum		0.37		
Daniel, Hirshleifer and Sun 3-factor model	Daniel, Hirshleifer, and Sun (2020)	Market, long-horizon and short-horizon mispricing factors		0.42		1.61**

The table outlines prominent asset pricing models from the factor literature, and lists the factors included in each model. Columns 1 and 2 indicate the name of the model and the scholarly article in which it was introduced. Column 3 lists the factor(s) used in that model. Column 4 reports the maximum Sharpe ratio (MS) results from Table 11 (Panel B) of Hou et al. (2015). Column 5 reports the maximum Sharpe ratio (MS) results from Table 5 of Hou et al. (2020a). Column 6 reports GRS F-Statistics and the corresponding significance levels for 73 anomalies, value-weighted, NYSE deciles from Table 5 of Stambaugh and Yuan (2017). Column 7 reports GRS F-Statistics and the corresponding significance levels for 34 anomalies from Table 7 of Daniel, Hirshleifer and Sun (2020). ***, ** and * represent significance at 1%, 5%, and 10% levels respectively

The Merton (1973) Intertemporal Capital Asset Pricing Model (ICAPM) and Ross (1976) Arbitrage Pricing Theory (APT) were offered as alternatives to the CAPM, highlighting the need for realistic assumptions. Ross (1976) popularized the term “factors”, and his APT lays the foundations for multifactor models. The APT expresses the expected returns on individual assets as linear combinations of the returns on one or several common factors capturing sources of risk that are priced in a no-arbitrage economy. In further studies, empirically motivated factors such as Basu’s (1977) price-earnings-based value factor and Banz’ (1981) size factor further document the insufficiency of CAPM to fully explain asset returns, calling for a more complex factor pricing model.

Addressing such concerns around the CAPM, Fama and French (1993) propose a 3-factor model combining market, size, and value factors that until recently has been the standard academic workhorse model to rationalize factor premia in equity returns. However, it does not explain the returns on price momentum factors, a strategy that buys stocks with high recent returns (looking back three to twelve months), and shorts stocks with low recent returns (Jegadeesh and Titman 1993). Consequently, Carhart (1997) proposes a 4-factor model by extending the Fama and French 3-factor model to include a one-year momentum factor alongside size, value, and the market portfolio.

Subsequent studies identify further regularities in stock returns that even the 4-factor model fails to capture, including quality factors such as investment and profitability put forward by Novy-Marx (2014) and Aharoni et al. (2013). Subsequently, Fama and French (2015) expand their 3-factor model to a 5-factor model by adding profitability (RMW) and investment (CMA) factors. By not including a momentum factor, they treat momentum as a “premier anomaly”, unexplained by the CAPM and their own model, even many years later. The two new factors, RMW and CMA, render the value factor redundant, suggesting the use of a more parsimonious 4-factor model with market, size, investment, and profitability factors alone. However, since value is one of the most sought-after factors among institutional investors, the use of the 5-factor model is warranted, and it essentially gives rise to the same abnormal returns as the 4-factor model. In a related vein, the Hou et al. (2015) q-factor model, which is based on investment theory, combines an investment factor, a profitability factor, a market factor, and a size factor. The authors find that the q-factor model outperforms the Fama and French 3-factor model and Carhart’s 4-factor model by capturing most of the anomalies that these two models fail to account for.

The growth in the number of mispricing-based factors has prompted the development of mispricing-based factor models. Instead of constructing a model based on single anomaly factors such as size or value, Stambaugh and Yuan (2017) suggest combining information across multiple anomalies and construct two mispricing factors by averaging across 11 well-accepted anomalies in order to obtain a less noisy measure of mispricing. They ultimately propose a 4-factor model by combining the two aggregate mispricing factors, labelled management and performance, with a size factor and a market factor. Similarly, in order to distinguish the two complementary aspects of mispricing, Daniel et al. (2020) develop a 3-factor model that features a market factor, a long-horizon factor (to capture long-term mispricing due to investor overconfidence), and a short-horizon factor (to capture short-term mispricing stemming from investor underreaction).

The significant growth in the number of suggested factors in the literature has intensified the search for an asset pricing model that identifies a parsimonious set of key factors useful in explaining the cross-section of asset returns, which would deal with the factor zoo from an investor perspective as well. For instance, Daniel et al. (2020) 3-factor model is not only parsimonious but also outperforms the profitability-based model of Novy-Marx (2014), the Fama and French (2015) 5-factor model, the q-factor model (Hou et al. 2015), and the mispricing model of Stambaugh and Yuan (2017) in capturing a wide range of anomalies. In contrast, the q5-model of Hou et al. (2020a), which augments the q-factor model by including an expected investment growth factor, has been shown by the authors to outperform all the factor pricing models identified so far: their empirical evidence suggests that the q5-model outperforms eight competing factor models including the Daniel et al. (2020) 3-factor model.

However, several studies emphasize the complexities in comparing different factor models. For instance, Barillas and Shanken (2017) discuss why simply comparing time series regression intercepts (or test portfolio alphas) across different factor models is insufficient as they might not be applicable for non-traded factors like consumption growth. They highlight the sensitivity of model rankings to the choice of test assets and suggest the use of the GRS (Gibbons et al. 1989) *F*-Statistic for comparing nested models. Acknowledging the challenges in comparing non-nested models, they point out that the best model in terms of a single performance metric might not be as good as one would expect, because the excluded-factor evidence from the best model might favor another model. Fama and French (2018) review different approaches used in the literature for comparing factor models and use the maximum squared Sharpe ratio to compare the CAPM, the Fama and French (1993) 3-factor model, the Fama and French (2015) 5-factor model, and their 5-factor model plus momentum. With more than 400 factors in the factor zoo, they highlight the issues surrounding the comparison of multiple combinations of factors, and argue that it would be almost impossible to identify the surviving factors from the factor zoo with the current statistical tests due to ‘clouded’ levels of *p*-values from overtly torturing the same data over and over again.

We illustrate the challenge in pinning down the best factor model by reporting selected results from Hou et al. (2015) (q-model), Hou et al. (2020a) (q5 model), Stambaugh and Yuan (2017) (3-factor model), and Daniel et al. (2020) (3-factor model) in Table 1. Columns (4) and (5) report the maximum Sharpe ratio (MS) from Table 11 (Panel B) of Hou et al. (2015) and Table 5 of Hou et al. (2020a), respectively. Column (6) reports the GRS *F*-Statistic, which tests whether the alpha of each of the anomalies tested is equal to zero, for 73 anomalies from Table 5 of Stambaugh and Yuan (2017), and column (7) reports the GRS *F*-Statistic for 34 anomalies from Table 7 of Daniel et al. (2020). Looking at the maximum Sharpe ratio reported by Hou et al. (2015) and Hou et al. (2020a) sees their q-factor and q5-models emerging as the best models. Whereas looking at In contrast, the GRS test-statistics in column (6) from Stambaugh and Yuan (2017) reveals that their 3-factor model has the lowest GRS *F*-statistic. Likewise, Daniel et al. (2020) report that their 3-factor model has the lowest GRS *F*-test statistic in column (7). Overall, the lack of a common test metric, set of test assets, and number of anomalies tested complicates direct comparison of metrics reported in published articles.

Some studies report the performance of several asset pricing models across a number of metrics. Ahmed et al. (2019) compare ten prominent asset pricing models and find inconclusive results. In their time-series tests, the Stambaugh and Yuan (2017) 4-factor model emerges as the best performer followed by the q-factor model. However, all tested models struggle to explain the returns on small stocks. In cross-sectional tests, the q-factor model, the Fama and French (2015) 5-factor model, the Fama and French (2015) 4-factor model, and the Barillas and Shanken (2017) 6-factor model perform best, followed by the Stambaugh and Yuan (2017) 4-factor model. Given the change in model rankings from different testing procedures, the authors caution that model comparisons are highly sensitive to the choice of test assets and comparison techniques.

In a nutshell, different combinations of factors can help to create powerful factor-based models capturing variation in expected returns without gaining exposure to unintended sources of risk and ensuring as much diversification of other sources of risk as possible. Whether the individual factors in such models stem from rational asset pricing theory, crude empiricism, or both, multi-factor models have become the dominant approach to explaining variation in expected returns, and such models can guide investors in their choice of factors.

### Evidence of cross-sectional return predictability across the globe

Investors across the globe have been keen to adopt factor-based investment strategies. According to the Invesco Global Factor Investing Study (2019), 50–60% of surveyed institutional investors in North America, Europe, the Middle East, and Africa (EMEA), and Asia–Pacific (APAC) intend to increase their factor allocations over the next three years. This is despite the fact that the research evidence underpinning factor investing is largely based on the US equity market, emphasizing the need to examine factor returns outside the US.

Calling it the “academic home bias” puzzle, Karolyi (2016) shows that only 23% of all empirical finance articles examine non-US markets. Given the convincing US evidence, there has been increasing academic interest in confirming the existence of factor premia in other regions. Despite limitations to data and breadth of non-US equity markets, the primary finding has been heterogeneity in the significance of many return predictors across regions. Still, some important predictors appear to work reasonably consistently across regions. For example, Haugen and Baker (1996) note a commonality in the primary return determinants across five major markets (US, Germany, France, UK, and Japan), especially prominent style factors such as value and momentum factors as noted below.

#### Value

There is extensive international evidence that various value factors on average generate positive equity return premia, especially in emerging markets. Chan et al. (1991) investigate returns to earnings yield, the book to market ratio, and cash flow yield in Japanese and US equity markets, documenting that these fundamentals strongly predict expected returns. Similarly, Capaul et al. (1993) find evidence for value premia in France, Germany, Switzerland, the United Kingdom, Japan, and the US. Fama and French (1998) also document value premia in 12 major developed international stock markets. They note the “hazardous” distributional properties of security returns in emerging markets, although a value factor based on the book-to-market ratio seems to work in 12 of the 16 emerging market countries in their sample. Rouwenhorst (1999) finds further evidence in support of a value factor premium in emerging markets. Similarly, studying 35 emerging markets between 1985 and 2000, Barry et al. (2002) offer strong emerging market evidence confirming the existence of a value premium.

#### Momentum

Another style factor that works across the globe is momentum, as documented in many international studies (see Rouwenhorst (1998) for European countries and Griffin et al. (2003) for global evidence). Rouwenhorst (1999) questions whether the same cross-sectional factors drive returns in developed and emerging markets, confirming slightly weaker momentum premia in emerging markets. However, more recent evidence of Griffin et al. (2010) suggests the opposite, with annual momentum profits averaging 8.7% in developed markets and 11.4% in emerging markets.

While momentum is a significant return factor in most markets, academic research has pointed out that momentum strategies fail to work in Japan. Asness (2011) argues that such evidence is not casting data mining doubts on international momentum effects. When viewing value and momentum factors as a single system, Japanese return behavior is consistent with the international evidence. These findings resonate with the universal profitability of value and momentum documented in Asness et al. (2013) and Fama and French (2012). Hou et al. (2011) also confirm that medium-term stock price momentum is priced in international equity markets and complements the value factor. Hence, the existence of value and momentum premia has been documented in several international markets.

#### Beyond value and momentum in international stock markets

Other factors have also been examined in global equity markets. Ang et al. (2009) find that a low-volatility strategy is profitable in 23 developed countries. Based on such findings for many equity markets across the world, researchers have concluded that there is no reward for bearing volatility risk, thereby strengthening the case for a low-volatility factor (Baker and Haugen 2012). Blitz et al. (2013) report that the low-volatility effect has become stronger due to delegated portfolio managers who tend to divert attention from low-risk stocks, with this impact being stronger in emerging markets than in developed markets. In a related vein, Asness et al. (2014) support the international equity market evidence of the betting-against-beta (BAB) factor. They dismiss the suggestion that industry bets drive BAB factor premia and document significant risk-adjusted returns to industry-neutral BAB portfolios across 49 US industries and in 60 of 70 global industries. The international evidence of different factors dispels concerns surrounding country-specific performance of factor strategies.

### Performance of equity factors across the globe

Translating paper returns into realized profits is an important concern of investors, especially outside of developed markets. Accounting for realistic constraints faced by institutional investors, Van der Hart et al. (2003) find that value, momentum, and earnings revisions are stronger predictors of returns than size and liquidity in 32 emerging markets. In further evidence focused on the dynamics of frontier markets relative to developed and emerging equity markets, De Groot et al. (2012) report evidence of a size premium in frontier markets that is not explained by exposure to global size, value, market, or momentum factors. They also find that value and momentum strategy returns survive transaction costs. Overall, these findings suggest that many style factors are profitable both in the United States and across the globe, underpinning the growth of factor-based investment strategies in global institutional portfolios.

In order to compare the magnitude of international equity factor premia, we gather empirical evidence on the salient factors in different regions between December 2001 and July 2020. While the academic literature often analyzes factor returns in ways that introduce practical caveats (most prominently the inclusion of microcap stocks and the use of equally-weighted portfolios), we focus on established factor indices provided by MSCI. They are a better gauge for the practical efficacy of equity factors around the globe since they employ realistic weighting schemes and focus on investible universes. The Appendix provides a more detailed description of the construction of the MSCI factor indices.

Table 2 reports performance for the equity factors Value, Size, Momentum, Quality, and Low Volatility alongside the corresponding market index returns. Panel A uses equity factors built for the MSCI All Country World Index (ACWI), while the other panels use factors for the United States (Panel B), Europe, Australasia, and the Far East (EAFE) (Panel C), and Emerging Markets (EM) (Panel D). The evidence is highly consistent across all regions. To benchmark the factor return indices, the annualized return for the MSCI ACWI within the sample period is 6.84% p.a. with a volatility of 15.4%. A corresponding index investment gave rise to a modest risk-adjusted return, as measured by a Sharpe ratio of 0.33. The maximum drawdown of 54.5% occurred in the global financial crisis (GFC) of 2008/2009.

**Table 2:** Equity factor performance around the world

	Return p.a. (%)	Volatility p.a. (%)	Sharpe ratio	Max DD %	Active return p.a	Tracking Error %	Information ratio
Panel A: Global							
Market	6.84	15.38	0.33	54.48			
Value	5.43	15.98	0.23	56.41	− 1.41	2.90	− 0.49
Size	8.18	17.49	0.37	58.55	1.34	4.73	0.28
Momentum	11.10	15.46	0.61	57.79	4.26	7.16	0.59
Quality	9.31	13.61	0.56	45.40	2.47	3.99	0.62
Low volatility	8.78	10.54	0.67	38.79	1.94	7.41	0.26
Panel B: US							
Market	8.37	14.38	0.46	48.04			
Value	6.47	14.86	0.32	51.74	− 1.90	3.81	− 0.50
Size	8.95	16.98	0.43	52.15	0.58	4.52	0.13
Momentum	11.62	14.45	0.69	50.21	3.25	7.38	0.44
Quality	9.81	13.04	0.62	37.98	1.44	3.63	0.40
Low volatility	8.36	11.26	0.59	40.64	− 0.01	6.03	0.00
Panel C: EAFE							
Market	6.04	16.33	0.29	56.00			
Value	4.98	17.54	0.21	57.82	− 1.06	3.09	− 0.34
Size	7.46	16.88	0.36	57.40	1.42	3.40	0.42
Momentum	8.43	15.39	0.46	56.35	2.39	7.42	0.32
Quality	9.12	14.94	0.52	47.88	3.08	4.92	0.63
Low volatility	8.28	11.68	0.60	41.70	2.24	7.27	0.31
Panel D: EM							
Market	9.33	21.03	0.36	61.91			
Value	8.57	21.26	0.32	59.85	− 0.76	2.87	− 0.26
Size	9.36	21.22	0.36	63.08	0.03	4.06	0.01
Momentum	13.32	22.05	0.53	68.54	3.99	6.92	0.58
Quality	10.95	19.79	0.47	58.44	1.62	4.32	0.38
Low volatility	10.90	16.64	0.55	52.24	1.57	6.06	0.26

The table reports the performance of MSCI style factor indexes across different geographies between December 2001 and July 2020. We compute annualized returns, volatilities, active returns (relative to the market) as well as corresponding tracking errors. Maximum drawdown gives the maximum loss suffered within the sample period. Panel A covers the performance of global factor portfolios as given by MSCI’s ACWI universe, representing large and mid-cap equity performance across 23 developed and 27 emerging markets. Panel B is for the US that represents the performance of the large and mid-cap segments and aims to represent ~ 85% of the US market. In Panel C, the MSCI EAFE Index is designed to represent the performance of large and mid-cap securities across 21 developed markets, including countries in Europe, Australia and the Far East, excluding the US and Canada. Panel D covers large and mid-cap securities across 26 Emerging Markets. For risk-free rates, Panel A, B and D use 3-month US LIBOR rates and Panel C uses 3-month EUR LIBOR rates as cash input. Details on data sources and variable definitions are provided in the Appendix

Active returns capture the factors’ performance contribution relative to the index benchmark. Table 2 shows that all factors outperform the market index except for Value. Value has an annualized active return of − 1.41% p.a. relative to the market and suffered a more severe drawdown in the GFC than the benchmark index. Despite earlier evidence suggesting that Value is a more procyclical investment style, it has continued to display weak performance in the second half of the sample period. While Momentum is similar to Value in terms of volatility and drawdown statistics, it has the highest return (11.1% p.a.) of all factors considered, thus outperforming the market by 4.26% p.a. This corresponds to a risk-adjusted active return of 0.59% p.a. (as measured by the information ratio capturing the active return per unit of risk relative to the benchmark portfolio).

The Quality factor is associated with similar risk-adjusted active returns (information ratio of 0.62), but represents a more defensive absolute risk-return characteristic: the volatility of Quality factor returns is 13.6%, and the maximum drawdown is almost ten percentage points lower than that of the market (45.4% versus 54.5%). The maximum drawdown is even lower for Low Volatility (38.8%), consistent with this investment style having a considerably lower market beta. Indeed, the Low Volatility factor displays the lowest volatility across all regions—its ex post volatility of 10.5% is around two thirds of global market volatility. Low Volatility nevertheless outperformed the market by 1.94% p.a. over the sample period and shows the highest Sharpe ratio among all global factors (0.67).

Empirical research has also studied the performance of factor strategies with lower implementation costs such as ETFs and mutual funds. In particular, Gelderen and Huij (2014) investigate the performance of prominent style factors such as low volatility, size, and value in US equity mutual funds. They not only evidence significant excess returns for these factor portfolios, but also find that the performance is persistent over time. In a related vein, Elton et al. (2019) document that combinations of factor ETFs outperform active US equity mutual funds most of the time. Still, real-world frictions may impact investors’ profitability especially when switching between factors or changing asset managers frequently. For example, Gelderen et al. (2019) find that despite style factors having a significant premium with a buy-and-hold strategy, rebalancing costs erode a significant portion of the factor profits. Hence, despite the convincing performance of factors, the final profit earned by investors is limited by real-world frictions.

### The advent of machine learning

#### Promises and pitfalls

Machine learning (ML) is a collective term that refers to using computer algorithms to infer meaningful patterns from a dataset. Depending on the selected hyperparameters, ML can be used to cater to both low- and high-dimensional setups, that is when one is facing only a few predictors or many of predictors. Increased data availability and computational capabilities have opened doors for ML algorithms in the investment management industry, and this class of techniques is increasingly used for return prediction and clustering of candidate factors. Approaching the factor zoo as a high-dimensional problem, ML appears to be a natural solution.

The attractiveness of ML techniques stems from their flexibility, distribution-free specification, and data-driven perspective. ML techniques have been used to construct portfolios with more accurate risk and return forecasts and under more complex constraints, to devise novel trading signals and execute trades with lower transaction costs, and to improve risk modeling and forecasting by generating insights from new sources of data (Bartram et al. 2020a). Other advantages of ML methods stem from their estimation procedure that allows joint testing of a large number of cross-sectional stock characteristics, focusing more on predictive accuracy and offering a framework to deeply exploit potential non-linear relationships (Freyberger et al. 2020).

Given the required technical skills, few researchers have attempted to apply ML techniques to testing the significance of different return predictors. Gu et al. (2020) exploit the ability of ML techniques to accommodate large numbers of predictors and capture potential non-linearities and predictor interactions. Based on 94 stock characteristics, they document high out-of-sample predictive R-squared for ML return forecasts, with liquidity, volatility, and price trends being the most significant predictors. They trace the predictive gains of the best performing models to their ability to capture non-linear predictor interactions missed by other classical statistical methods. Similarly, Bianchi et al. (2020) use machine learning methods for predicting bond excess returns. Based on more than 100 macroeconomic and financial variables, yields included, the authors document higher out-of-sample R-squared compared with more traditional econometric methods.

As the ultimate goal of factor investing is to cater to the investor’s risk-return objectives, newer ML techniques have been explored to automate portfolio construction. To this end, Feng et al. (2019) utilize 62 firm characteristics as inputs to train a deep learning model for US equities. Augmenting the Fama–French 3-factor model with factors identified by the deep learning model, they document marginal improvements in the R-squared in the time series analysis of portfolio returns, but impressive out-of-sample performance in cross-sectional returns prediction. Such encouraging results validate the scope of artificial intelligence and ML techniques in factor investing.

ML methods have also helped to uncover weaknesses of existing factor models in dealing with the factor zoo (Freyberger et al. 2020). Its multi-dimensionality calls for models that can identify incremental information in each characteristic to eliminate the factors that are subsumed in joint tests and ultimately identify the surviving factors. Furthermore, existing models do not consider nonlinear relationships between characteristics and returns (Fama and French 2008), prompting Cochrane (2011) to suggest the usage of different techniques to overcome such limitations. Kozak et al. (2020) use ML techniques to investigate 120 return predictors and find that traditional 3-factor (or even 5-factor) models are insufficient to explain portfolio returns in a high dimensional setup.

Using 36 well-known return-predictive characteristics, Freyberger et al. (2020) find that a linear model selects 21 characteristics, while non-linear models select only 8, but increase Sharpe ratios by 50% out-of-sample. Their results are robust to the choice of tuning parameters, addressing data mining and overfitting concerns. Hence, at a minimum, ML techniques could help identifying surviving factors in the factor zoo. Feng et al. (2020) stress the importance of choosing the correct benchmark for navigating the factor zoo and propose a model framework to select factors from a list of candidates. The improved framework aims to identify fewer significant factors that add value after controlling for the three Fama–French factors. Thus, it appears that ML techniques may help reduce the dimensionality of the factor zoo, albeit while introducing new complications and challenges.

Sceptics consider ML in asset pricing a hard bargain, however. Because ML techniques are purely driven by the specific data used for analyses, they are susceptible to data mining and overfitting. Overfitting occurs when the ML model learns the training data too well and thus may fail to work with a new dataset. Researchers have suggested that the ratio of the degrees of freedom to the number of observations in the dataset could reflect the extent of overfitting in the model. As examining the factor zoo would require joint testing of hundreds of characteristics, the large number of independent variables would imply very high degrees of freedom, potentially leading to overfitting the training dataset.

The underlying ML mechanisms are often perceived as a “black box” with questionable theoretical underpinnings. From an institutional investor perspective, the inability to attribute investment performance can render client communication a challenge. Avramov et al. (2021) question the interpretability of signals derived from ML techniques and critically evaluate the contributions of ML techniques in return prediction. They find a steep decline in return predictability of ML techniques after excluding microcaps or distressed firms and adjusting for market states. ML strategies are particularly successful in specific market states, such as periods of high investor sentiment or high market volatility. ML strategies tend to have higher turnover, and hence higher implementation costs, further emphasizing the need to approach such complex techniques with caution. Borghi and de Rossi (2020) estimate a series of models along the lines of Gu et al. (2020) and apply trading constraints when optimizing the portfolio, i.e. they limit turnover and the amount traded in each stock based on its average daily (trading) volume. While performance deteriorates, the conclusion that ML is superior to traditional alternatives at combining factors is unchanged.

In a related vein, Leung et al. (2021) investigate the potential of ML techniques for predicting the cross-section of stock returns. Using a set of 20 stock characteristics in an investible global stock universe, they confirm that ML forecasts are statistically superior to those based on standard linear models. Yet, this advantage is driven by exposure to hard-to-arbitrage factors such as short-term reversal, raising doubts about the economic relevance of ML models for practical institutional investment. Indeed, the added value in real-world portfolio simulations is less pronounced and depends heavily on the ability of an investor to take risk and implement trades efficiently.

#### Textual factors

Novel sources of information have been exploited by researchers and practitioners to identify newer sources of return predictability by using state of the art techniques. Natural language processing (NLP) has become an important methodology for extracting information from unstructured textual data sources. NLP has found its way into factor investing studies to extract return predictors from published financial disclosures and related materials such as 10-K filings or earnings call transcripts. NLP techniques search for patterns in financial narratives to infer properties such as sentiment or obfuscation in the words that corporate executives use in their disclosures and communications with the market. For example, in inferring executives’ sentiment, financial narratives might be classified into broad groups such as positive, negative or neutral sentiment. As investor sentiment can be used as a short-term return predictor, such information could be useful during portfolio rebalancing.

Surveys of text mining in the broader field of accounting and finance highlight the information content hidden in corporate disclosures that can help predict future firm performance (Li 2008; Kearney and Liu 2014; El-Haj et al. 2019). Quantitative data carry more easily interpretable information than qualitative data, while the complex and ambiguous nature of oral and verbal communications could limit the efficiency of even the most advanced text mining tools. To this end, custom dictionary techniques and topic modeling are emerging as potentially more powerful approaches. Dictionary methods use the frequency of occurrence of a list (or bag) of words as a measure (see e.g. Bartram et al 2011). The limited range of commonly used dictionaries and the equal weighting of all occurrences of a word in different contexts, however, raises concerns about the reliability of such methods (Hansen et al. 2017).

In contrast to dictionary-based techniques, topic modeling techniques focus on uncovering the underlying semantic structures by recognizing topics that occur in a collection of documents. The most prevalent topic modeling technique is Latent Dirichlet Allocation (LDA) proposed by Blei et al. (2002). LDA approaches a document as a set of different topics and then measures the dominance of each topic. To this end, Israelsen (2014) uses risk factors extracted from 10-K filings for style analysis and offers risk-based explanations for the existence of market, size, value, and momentum premia, illustrating the importance of qualitative information in firm disclosures. Topic modeling can also be useful in the development of text-based multi-factor models. Using LDA to uncover the risks disclosed in a firm’s 10-K filings, Lopez-Lira (2019) identifies four systematic factors (technology, production, international, and demand) that help explain the cross-section of returns. This text-based 4-factor model has the smallest GRS *F*-statistic compared with the Fama and French (2015) 5-factor model, Stambaugh and Yuan (2017)’s mispricing-based factor model, and the Hou et al. (2015) q-factor model. However, a possible downside with topic modeling is that different researchers may end up identifying different topics that are inherently subjective, rendering the findings non-replicable. Hence, investors and portfolio managers need to be cautious when using such techniques for factor selection.

## Factor investing beyond equities

A majority of the factor investing literature focuses on equities, perhaps reflecting the absence of a clear theoretical consensus on how best to identify and model drivers of equity risk and return, but also greater investor interest and the relatively rich and diverse data available for equities compared with other financial asset classes (Bartram and Dufey 2001). Nevertheless, in recent years researchers have increasingly attempted to apply insights from equity factor research to other asset classes such as currencies, fixed income, and commodities. For instance, Asness et al. (2013) find evidence for the existence of value and momentum premia in currencies, government bonds, and commodities, as well as equities. Similarly, Koijen et al. (2018) provide evidence for carry trade return predictability in global equities, global bonds, currencies, commodities, US Treasuries, credit, and equity index options. This section summarizes some of the recent evidence for the main non-equity asset classes, highlighting similarities and differences relative to findings for equities.

### Currencies

Currency factor strategies are used by institutional investors both for hedging unwanted currency exposures in internationally diversified portfolios and as a stand-alone investment asset class. Researchers have noted a tendency for currency fund managers to load on standard currency style factors, such as carry, value, momentum, and volatility (Pojarliev and Levich 2008). In addition to carry, value and momentum, recent research has identified several other related factors including dollar exposure, dollar carry, factors based on macro-economic fundamentals such as output gap and the Taylor rule (Bartram et al. 2020b), global external imbalance (Corte et al. 2016), and business cycle factors that identify strong and weak economies (Riddiough and Sarno 2018).

#### FX carry

Based on early research establishing that uncovered interest rate parity (UIP) does not hold (Bilson 1981; Fama 1984), the FX carry factor seeks to exploit the interest rate differentials of high- and low-yielding currencies. Hansen and Hodrick (1980), Bilson (1981), and Fama (1984) address the failure of UIP in the context of the forward premium puzzle and hence can be thought of as academic precursors for carry trading in foreign currency markets. The carry trade as a cross-sectional investment strategy involves borrowing in low-interest rate currencies and investing in high-interest rate currencies. While currency movements according to UIP should negate the resulting profits, this is empirically not the case, rendering FX carry investments profitable.

Carry trades appear to be sensitive to market movements and have experienced severe crashes with extreme drawdowns of about 30% (Doskov and Swinkels 2015). Consequently, it has been suggested that positive returns from carry could provide compensation for crash risk (e.g., Brunnermeier et al. 2008; Farhi et al. 2009). Brunnermeier et al. (2008) refer to the carry trade as “going up the stairs and down the escalator” and find that carry trade unwinding happens during liquidity squeezes and periods of heightened FX volatility (see also, Menkhoff Sarno et al. 2012a). Bhansali (2007) documents a positive relation between carry trade payoffs and currency volatility and concludes that currency carry trades perform best in low-volatility regimes. Carry trade strategies in other major asset classes also seem to perform poorly during recessionary periods (Koijen et al. 2018).

To further rationalize the existence of carry premia, Lustig and Verdelhan (2007) and Lustig et al. (2011) show that US consumption growth explains a significant portion of carry trade returns, arguing that carry trades reflect compensation for bearing the risk of a large depreciation during global downturns (Hoffmann and Studer-Suter 2017). Relatedly, the peso problem is a commonly offered explanation for carry trade performance, i.e. it is argued that investors are compensated for exposure to relatively rare events that have extreme negative outcomes such as currency devaluations (Burnside et al. 2011). However, Jurek (2014) rejects peso problem explanations for the outperformance of carry trades and finds that negative skewness is priced in the cross-section of carry trades.

#### FX momentum and FX value

Momentum strategies have also been found to be profitable in currency markets (Menkhoff et al. 2012b). Although FX momentum strategies require frequent rebalancing and hence higher transaction costs, they are an important diversifier to counteract the downside risk of carry trades (Barroso and Santa-Clara 2015). With carry trade positions being unwound in times of crises, currency momentum-based trading will quickly anticipate such currency movements and effectively establish positions offsetting carry currency allocations.

Potentially complementing currency momentum as a short-term hedge, currency value strategies identify overvalued and undervalued currencies in order to gain exposure to expected long-term reversal of currencies to their fundamental values (Menkhoff et al. 2017). Asness et al. (2013) use the 5-year change in purchasing power parity (PPP) to compute currencies’ value and document significant value and momentum premia for a sample of G10 currencies between 1979 and 2011. In computing value factors for currencies, Baku et al. (2020) employ alternative proxies for fundamental value including PPP, REER (real effective exchange rate), and FEER/BEER (fundamental/behavioral equilibrium exchange rates). They find that emerging market FX value factors based on PPP and FEER/BEER have higher Sharpe ratios than their G10 counterparts. Similarly, FX carry and momentum factor returns are stronger in emerging market currencies than those in developed market currencies.

Bartram et al. (2020b) combine 11 predictors of currency excess returns into a combined mispricing measure documenting higher signal to noise ratios compared with individual factors. Fast decay of predictor ranks and performance as well as evidence of significant returns after comprehensive risk-adjustments challenge risk explanations to currency trading strategies. The mutual diversification benefit of combining carry, value, and momentum factors has been repeatedly confirmed in the literature (Ranganathan et al. 2020); in addition, such factors meaningfully expand the investment opportunity set of international multi-asset investors (Kroencke et al. 2014).

### Fixed income

Fixed income markets are characterized by comparatively lower liquidity, and tradable securities are more heterogeneous with different coupons, maturities, and covenant structures. Perhaps as a consequence, there is less corresponding factor investing research. From a top-down perspective, fixed-income investors are exposed to credit and interest rate risks, yet style factors based on duration, carry, quality, and low-volatility based definitions have been proposed to manage rates. However, despite difficulties in controlling for the pricing implications of contract design features, factors pertaining to corporate bonds, also referred to as credit factors, have recently become popular among practitioners, especially given the large cross-sectional universe of corporate bonds available for analysis.

#### Corporate bond factor investing (Credit factors)

An early study by Hottinga et al. (2001) explores the vast fixed income market and the promising scope of factor investing strategies in corporate bonds. Various style factors based on corporate bond characteristics, often paralleling those in equity markets, have been documented as significant predictors of bond returns. Specifically, Bai et al. (2019) show that downside risk, credit risk, and liquidity risk are priced in the cross-section of corporate bond returns and confirm that these three risk factors are not subsumed by other bond market factors. Moreover, momentum, low-volatility, and quality have been documented as significant predictors of bond returns (Israel et al. 2018); Bali et al. (2020a, b) document long-term reversals in the corporate bond market. Kelly et al. (2020) propose a new conditional factor model for individual corporate bond returns based on instrumented principal components analysis. Brooks et al. (2020) find that exposure to traditional risk factors largely explains the active returns of fixed income managers.

In addition to bond factors, a number of equity factors such as size, profitability, and asset growth also have predictive power for bond returns (Chordia Goyal et al. 2017; Jostova et al. 2013; Gebhardt et al. 2005). Bektić et al. (2019) find that the Fama and French (2015) size, value, profitability, and investment factors have explanatory power for returns of US high yield corporate bonds, but the relations are less pronounced for US and European investment grade bonds. Avramov et al. (2019) show that investor sentiment and financial distress jointly drive bond and equity overpricing underlying market anomalies. However, according to Choi and Kim (2018) some factors (e.g., profitability and net issuance) fail to explain bond returns, while for others (e.g., investment and momentum) bond return premia are too large compared with their loadings, or hedge ratios, on equity returns of the same firms. Moreover, Bali et al. (2020a, b) show that while equity characteristics produce significant explanatory power for bond returns, their incremental predictive power relative to bond characteristics is economically and statistically insignificant when using machine learning.

#### Government bond factor investing (Rates factors)

Style factors such as carry, value, momentum, and defensive also manifest in the cross-section of global government bonds, albeit deriving from a relatively small sample of sovereign bond rates. Brooks and Moskowitz (2018) analyze yield curve premia and conclude that carry, value, and momentum factors better explain their cross-sectional and time-series variation than the underlying principal components. Beekhuizen et al. (2019) provide a thorough analysis of (yield curve) carry strategies that involve selecting maturity buckets with the highest units of carry. While the basic premise of the carry trade is borrowing a low-yielding asset to invest in a high-yielding asset, carry trades in bonds are designed to capture the roll yield, which is the price increase when the longer-term bond rolls down the yield curve.

The curve carry factor for bonds uses the slope of the yield curve directly by going long on a longer maturity, say a 10-year bond, and short on shorter maturity, say a 2-year, bond. Beekhuizen et al. (2019) find that the curve carry strategy subsumes the defensive betting-against-beta strategy that invests in the shortest maturity buckets. Brooks et al. (2018) investigate rates factors and find that combining styles including carry, value, momentum, and defensive deliver a Sharpe ratio close to one over 20 years of data. The authors emphasize the appeal of such rates factors, given that they have low sensitivity to macroeconomic variables and thus diversification benefits. In a related vein, Kothe et al. (2021) show how rates factors expand investors’ opportunity set and can benefit their tail-hedging or return-seeking investment objectives.

### Commodity style factors

Commodities find their way into institutional investor portfolios as they are typically thought of as an alternative asset class offering protection against rising inflation, in addition to offering diversification benefits because of their low correlation with traditional asset classes including equities and bonds. However, the 2008/2009 Global Financial Crisis and the 2020 Covid-19 Crisis saw commodity prices fall in tandem with other asset classes, raising questions about the diversification benefits of commodities (Bartram and Bodnar 2009). Still, there is ample evidence of predictability in the very heterogeneous cross-section of commodities, and commodity factors help to broaden investors’ opportunity set (Giamouridis et al. 2017; Blitz and De Groot 2014). Similar to other asset classes, commodity returns can be explained by commodity style factors, including carry and momentum.

Miffre (2016) and Sakkas and Tessaromatis (2020) both present overviews of relevant commodity factors that typically build on commodity fundamentals such as term-structure variables and past price momentum. Miffre and Rallis (2007) find evidence for the existence of both short-term momentum and long-term momentum in commodities, while noting that momentum effects are not predicted by extant asset pricing models. With carry and momentum playing a role in predicting commodities returns, it seems important to identify the fundamental economic drivers of commodity factor premia (Erb and Harvey 2006). Bakshi et al. (2017) show that a 3-factor commodity pricing model with market, carry, and momentum factors summarizes the cross-section of commodity returns better than 1-factor or 2-factor models. Hence, multi-factor models also appear relevant for commodities (Fernandez-Perez et al. 2018). In line with this conclusion, Hammerschmid and Lohre (2020) integrate time-series predictors and cross-sectional characteristics in a parametric portfolio policy context for commodity futures. Their final choice of three variables for the multivariate timing policy and six fundamental factors for the multivariate tilting portfolio outperforms an equally weighted benchmark.

### Multi-asset multi-factor investing

**Fig. 3:**
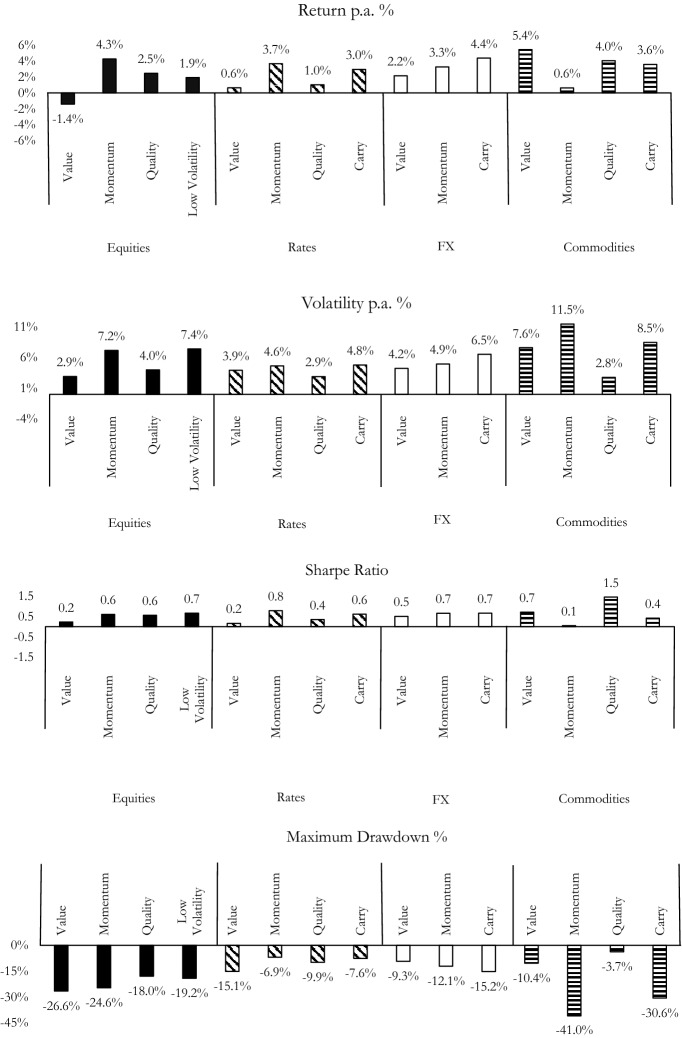
Style factor performance across asset classes. The figure depicts different performance metrics of style factor performance across equity, rates, FX, and commodities. Details on data sources and variable definitions are provided in the Appendix, see Table 3 for the underlying style factor indices. The maximum drawdowns indicate the maximum observed loss and are reported as negative to indicate the downside risk. The sample period is December 2001 and July 2020

Institutional investors do not necessarily consider different asset classes in isolation but may combine factors in multi-asset or cross-asset investment frameworks. To this end, Fig. 3 compares statistics on style factor performance across asset classes, namely excess returns, volatility, Sharpe ratio and maximum drawdown. All of the presented performance statistics refer to excess returns of long-short factor portfolios. Except for equity value, all style factors display positive excess returns throughout the sample period, thus reinforcing the validity of this investment paradigm.

While the average volatility across factors is around 5%, the least and the most volatile factors emerge within commodities; commodity quality comes in at some 3% volatility whereas commodity momentum has over 11% volatility. Importantly, many style factors deliver attractive risk-adjusted returns as measured by Sharpe ratios as well (note that the underlying factor indices account for transaction costs). While Sharpe ratios refer to the compensation of volatility risk, some style factor strategies come with considerable tail risk. Maximum drawdowns in Fig. 3 are often below −15% for the non-equity factors, with commodity momentum and commodity carry showing maximum drawdowns below −30%. Style factor returns tend to have low correlations, and their tail risk events typically do not coincide (Chambers et al. 2018). Consequently, embracing factor investing in and across different asset classes suggests ample diversification benefits for multi-asset multi-factor strategies. Indeed, the related literature typically suggests risk-based allocation schemes to harvest the associated premia in a balanced fashion (Dichtl et al. 2021).

## Rationalizing factor returns

The case for including specific factors in an investment strategy is undoubtedly strengthened if the role of fundamental economic factors in driving factor performance can be identified. However, it can often be difficult to distinguish empirically between risk and mispricing explanations of factor performance, especially when results are sensitive to the choice of asset pricing (or risk) model as the benchmark for expected returns. Beyond this challenge, recent research has emphasized the importance of excluding the possible role of data snooping biases as explanations for statistically significant factor returns, especially when theoretical support for a returns predictive relationship is weak. The following subsections outline how researchers are seeking to categorize factor premia with these considerations in mind.

### Why do factor premia exist?

The proliferation in the number of published return predictors in the literature highlights the need for having strong underlying rationales. Classic explanations include risk, mispricing, and statistical bias. Behaviorists argue that factor premia stem from persistent pricing errors, while supporters of rational pricing theories suggest risk-based explanations. In particular, behavioral explanations of factor premia posit that return predictability based on public information results from investors’ collective behavioral biases. For example, Lakonishok et al. (1994) reflect a behaviorist perspective in arguing that the value premium arises because under bounded rationality, investors tend to extrapolate past performance, thereby causing pricing errors. Hence, when a reversal happens, out-of-favor value stocks outperform seemingly glamorous stocks, resulting in the value premium (see, e.g. Haugen 1995).

In general, irrational investor behavior can result in market inefficiencies, oftentimes explaining mispricing by either over-reaction or under-reaction by investors to public information. However, a significant challenge to behavioral explanations is whether there are effective limits to arbitrage. Unless there are significant limits (or costs) to arbitrage, factor returns reflecting behavioral biases should disappear over time as investors with arbitrage capital take advantage of other investors’ biases. For assets traded in developed, liquid markets where well-informed institutional investors are active, limits to arbitrage are unlikely to be a primary explanation of the predictability of many factors.

In contrast, if factor premia exist as compensation for risk, then they should be persistent over time. The most prevalent argument is that factor premia compensate for risk that the CAPM fails to account for (Fama and French 1993, 1996). Berk (1995) is an early exponent of the idea that characteristic-based factors capture cross-sectional variation in discount rates due to unmodeled risk. He argues that holding future cash flows constant, smaller firms with lower market capitalizations have higher discount rates. Higher discount rates, in turn, imply higher expected returns. This argument can be extended to value factors constructed using market capitalization, or on a per share basis stock price, as a deflator. Fama (1998) interprets the global value factor as a risk premium, which is priced via discount rates when markets are efficient. However, whether discount rates and expected returns are higher due to (omitted) risk factors or investor mistakes is challenging to ascertain empirically.

Similar debates about the source of factor premia also occur in the literatures relating to other asset classes. To illustrate, Brunnermeier et al. (2008) suggest skewness or crash risk as an explanation for the FX carry trade. In contrast, Froot and Thaler (1990) favor a behavioral explanation and dismiss risk-based explanations for the forward discount bias, the key driver of carry trades. Before discussing means to disentangle the likely driver of a given factor, be it risk or mispricing, we first explore more broadly the various risk-based explanations that have been proposed.

#### Risk-based explanations for rationalizing factor premia

Investors will be keen to understand the potential risks they might be exposed to when engaging in factor investing strategies. Distress risk is often used to rationalize factor premia. For instance, Chan and Chen (1991) argue that the size effect is primarily driven by firms in distress, characterized by low profitability, high financial leverage, and low dividends. As such variation in returns is not captured by market returns, investors with exposure to size may simply be compensated for taking on distress risk. Fama and French (1996) report similar findings for the role of distress risk in explaining the value premium; they note that firms with high book-to-market ratios exhibit more uncertain future earnings. In a related vein, Kapadia (2011) finds that HML predicts firms’ future failure rates, suggesting that the value premium arises from investors requiring compensation for bearing financial distress risk. Finally, distress risk has also been linked to explanations for momentum. For example, Avramov et al. (2007) find that the profitability of momentum strategies is driven by firms with high credit risk; similarly, Agarwal and Taffler (2008) suggest that momentum is related to bankruptcy risk.

Further arguments in the literature focus on return-predictive factors being correlated with other sources of priced risk. For example, Campbell and Vuolteenaho (2004) suggest that the value premium represents compensation for bearing cash flow risk, because value firms pay out a greater proportion of capital as dividends and hence have higher book/market ratios. Consequently, value investors face higher exposure to cash flow risk. Related arguments concern duration-based explanations of the value premium. Lettau and Wachter (2007, 2011) and Schröder and Esterer (2016) show that growth stocks have higher future cash flows and cash flow growth. This manifests as higher equity duration (Dechow et al. 2004). In turn, longer-duration equities have higher discount rate risk exposure. Gormsen and Lazarus (2020) find evidence of premia related to duration for five equity factors, including value, profitability, investment, low risk, and payout. Guo et al. (2009) suggest that value premia reflect intertemporal pricing due to strong countercyclical variations in expected value premia. Hence, the value premium tends to be high during recessionary phases and low during expansionary phases. In a related vein, Andronoudis et al. (2019) use an ICAPM framework to show that R&D intensity, which also appears to attract a premium, is associated with higher equity duration and higher discount rate betas.

Operating leverage has also been associated with the value premium in equities. Donangelo (2020) uses a production-based asset pricing model to explain the role of labor leverage in the value premium. Using firm-level labor shares as a proxy for operating leverage, he finds a positive relationship between labor share and firms’ book-to-market ratios. Firms with high labor share are more exposed to priced risk, and thereby offer higher risk premia to investors.

Aretz and Pope (2018) also adopt a production perspective, through a real options model that values capital assets as a portfolio of production options. As a result of capital investment being costly to reverse, firms invest conservatively, but nevertheless can ex post have higher levels of assets in place than is optimal based on observed uncertain demand. The optionality elasticities of investment and production options causes the betas of equities to depend on the past history of demand and investment decisions. Aretz and Pope (2018) show that a measure of capacity overhang captures the optionality of equities and is a strong predictor of returns that helps explain momentum and profitability factors in pricing the cross-section of equities.

Disaster risk is less commonly studied, but it is another potential reason for the existence of factor premia (Rietz 1988). Following Barro (2006), Barro et al. (2009), Gabaix (2012), and Berkman et al. (2011, 2017), Siriwardane (2013) examines the links among value, size, and momentum premia and disaster risk, finding that the latter plays a role in explaining the cross-section of returns of the corresponding portfolios. The limited research in this area may reflect the inherent rarity of such events. Other risks that have been related to factor returns include illiquidity risk, inflation risk, country risk (Zaremba 2016), economic risk, such as changing volatility (Lettau et al. 2008) and income inequality (Gollier 2001; Hatchondo 2008), and political risk due to policy uncertainty (Pastor and Veronesi 2012) or unstable governments (Lam and Zhang 2014).

#### Delineating risk and mispricing

To empirically distinguish between alternative rationales for factor premia, scholars study their out-of-sample predictive performance (McLean and Pontiff 2016; Linnainmaa and Roberts 2018; Bartram et al. 2020b). This research compares factor returns in an original estimation sample period (“in-sample period”), the period between the end of the sample (in which the factor was identified) and the posting of the paper on SSRN (“out-of-sample period”), and the period after posting on SSRN (“post-publication period”) in order to uncover trends in cross-sectional return predictability. The idea is that any factor performance due to statistical biases is likely to disappear outside the original sample period. Similarly, factor returns that reflect mispricing are expected to decay or disappear in the post-publication period, if sophisticated investors seek to arbitrage the revealed predictability. Conversely, publication should not affect factor payoffs that are compensation for risk if assets remain fairly priced given that risk.

In this vein, McLean and Pontiff (2016) and Bartram et al. (2020b) reject risk-based explanations in favor of mispricing-based explanations by documenting a significant decrease in post-publication profits of many anomalies in equity and currency markets, respectively. For equity markets, the empirical evidence shows a 58% reduction of anomaly returns after publication (McLean and Pontiff 2016) and in recent years due to increased trading activity of hedge funds and lower trading costs (Chordia et al. 2014). The return decay is larger for predictors with lower arbitrage costs. McLean and Pontiff (2016) also report significant correlations between yet-to-be published predictors, although such relatedness decreases after publication. In contrast, Jacobs and Müller (2020) suggest no decay of factor performance for stock markets outside the United States. Such contradictory evidence within the US and across the globe calls for further examination of post-publication factor performance.

Since performance in out-of-sample and post-publication periods could both be affected by statistical bias, Linnainmaa and Roberts (2018) investigate pre-estimation sample period returns for 36 factors. They find that many factors, including profitability and investment, are significant for the in-sample period (1970–2004), but are insignificant in the pre-sample period (1926–1969). In a similar research setup, Wahal (2019) extends the sample back to 1926 and finds evidence for the existence of the profitability factor but not for the investment factor. This evidence is consistent with data-snooping biases for most factors, although alternative explanations such as changing macroeconomic regimes cannot be ruled out.

While research has traditionally interpreted risk-adjusted returns as evidence of mispricing, this interpretation critically depends on the validity of the risk model. To this end, recent research questions interpretations of reductions in factor premia as evidence of mispricing, suggesting that the evidence could also be consistent with time-varying compensation for risk. For instance, Kelly et al. (2019) develop an instrumented principal component analysis (IPCA) allowing for latent factors and time-varying factor betas. Their method introduces observable characteristics as instruments for unobservable dynamic factor betas. In evidence based on US equity market data, only 4 of 37 anomalies have IPCA alphas that are significantly different from zero, suggesting that many anomaly factors documented in the literature capture time-varying risk premia as opposed to reflecting market inefficiencies. However, Bartram and Grinblatt (2021) show that trading global stocks based on a regression-based measure of mispricing yields significant risk-adjusted returns. This holds true when controlling for traditional factor models (including all 50 factors from the Fama–French data library or their own 80-factor model). It also holds when using IPCA to control for time-varying risk premia even tied to mispricing itself.

Researchers have also employed other techniques to distinguish mispricing from risk-based explanations. Shleifer and Vishny (1997) argue that the returns of mispricing anomalies should be significantly higher for stocks with higher limits to arbitrage, such as those with smaller market capitalization and lower institutional ownership. Stambaugh et al. (2012) document an increase in mispricing-based anomaly returns during high-sentiment periods. This implies the existence of mispricing due to overpricing, which is exacerbated by short-selling constraints. However, consistent with Berk (1995), higher prices may also be consistent with lower discount rates, and this observation leads proponents of sentiment-based explanations for factor returns to distinguish between variation in sentiment as a behavioral phenomenon and the variation in discount rates as an economic phenomenon.

Beyond the existence of significant risk-adjusted returns, relatively fast decay of signal ranks and performance are also more consistent with a mispricing explanation for factor premia than with risk, as evidenced for the agnostic mispricing measure introduced by Bartram and Grinblatt for US (Bartram and Grinblatt 2018) and global equities (Bartram and Grinblatt 2021), and for currency predictors (Bartram et al. 2020b). The study of book-to-market effects in corporate bond returns may also aid in the understanding of why it influences asset returns more generally, since the future cash flows of corporate bonds, particularly senior bonds, are far less risky than for equity (Bartram et al. 2020c). Consequently, bond price movements have to arise largely from discount rate variation rather than from changes in projections of future cash flows. Nevertheless, delineating between risk and mispricing explanations of return predictors remains a challenge, and more powerful tests are required to distinguish between these competing explanations for factor returns.

### The dangers of data mining

Rationalizing a given factor’s efficacy through either risk or mispricing explanations is only a meaningful exercise if factor performance is not statistically spurious to begin with. Of course, the collective efforts of a multitude of academic and practitioner researchers scanning the limited datasets available to them for significant patterns of return predictability suggests that false positive, and hence spurious, in-sample results are to be expected. Such collective data mining raises concerns about the out-of-sample success and thus the usefulness of return predictive factors. To mitigate data mining concerns requires more rigorous empirical testing, including out-of-sample analysis, controlling for the statistical effects of multiple hypothesis testing on the same data, as well as plausibility checks based on well-defined theoretical economic priors.

#### Early studies accounting for data snooping

As computing power has grown rapidly, the risk of data mining (or snooping) has become more pronounced. Despite being recognized as a potential problem nearly a century ago (Cowles 1933), concerns about data snooping biases have only impacted the asset pricing literature quite recently. One widely cited exception is Lo and MacKinlay (1988) who caution against the increased likelihood of data-mined results with the increase in the number of publications in any given field. In related research, Lo and MacKinlay (1990) stress the potential importance of data-grouping techniques in determining the performance of return predictors by documenting a significant difference between tests of data-driven models and theory-driven models.

To illustrate the danger of identifying spurious factors, Ferson et al. (1999) show that a simulated alphabetically-sorted portfolio earns excess returns mimicking value premia, despite there being no obvious connection between the first letter of a company’s name and its expected return. This example illustrates the importance of a sound economic foundation, not just statistical significance in quantile portfolio spreads. Patton and Timmerman (2010) further emphasize the importance of examining the expected returns of all portfolio quantiles when developing a trading strategy. Instead of examining the performance of extreme quantile portfolios that is common in empirical asset pricing research, they recommend that researchers should test for monotonicity of returns across all quantiles to provide greater support for systematic relations between future returns and the sorting attribute. As observed by Romano and Wolf (2013), this test may not work if expected returns follow a non-monotonic relation or are weakly increasing, and the authors provide an alternative test that is immune under such circumstances.

Researchers have also identified more subtle channels through which data mining may manifest. Sullivan et al. (1999) explain the dangers of reusing the same data for inferences or model selection in an attempt to achieve satisfactory results. Using bootstrapping techniques, they perform a reality check on 7,846 trading rules and find that their best in-sample trading rule (the five-day moving average rule) is an insignificant predictor out-of-sample. In a related paper Sullivan et al. (2001) draw attention to the danger of using the same data for formulating and testing a hypothesis, thereby inadvertently increasing the chances of data mining. By using 100 years of daily data and bootstrapping techniques, they test calendar anomalies that do not have a strong theoretical motivation. The evidence suggests that calendar rule-based strategies such as the Monday effect are not as significant as originally suggested.

Overall, while individual researchers might limit their research to one or a small number of factors, data snooping biases will arise to the extent that an individual researcher retests multiple specifications for the same underlying construct, e.g., different definitions of value or momentum. Similarly, data snooping bias arises at the aggregate level as a result of different researchers investigating different potential factors but using the same data. This body of research suggests that it is important to control for data-snooping biases in evaluating claims that a factor successfully predicts asset returns.

#### Statistical methods to mitigate data snooping concerns

To account for multiple testing, the statistical literature advocates controlling for the family wise error (FWE) or the false discovery proportion (FDP). The FWE represents the probability of making at least one false positive (type I) error in the family of tested factors. The FDP is a less demanding test focusing on the number of false positive results within the set of positive results. Bonferroni’s (1936) well-known test to control for the FWE involves a *p*-value adjustment that divides the significance level by the number of hypotheses to be tested. Obviously, given the typically low single digit *t*-statistics obtained in return predictive regressions, if one tested the significance of hundreds of factors (as researchers have done collectively), one would reject the statistical significance of most if not all factors if one applied this adjustment. Fortunately, the literature has developed more powerful tests, such as the StepM-method of Romano and Wolf (2005) that incorporates the dependence structure of test statistics. Leippold and Lohre (2012a, b) are early adopters of such methods in accounting for multiple testing when investigating market anomalies across the globe. They provide an implicit proof of concept of the method’s power by documenting the robustness of momentum factors but not accruals factors using a battery of multiple testing procedures.

Harvey et al. (2016) implement the FDP testing framework to re-examine cross-sectional return patterns in equities. Of the 316 factors tested, they find between 80 and 158 false discoveries depending on the choice of statistical test. Taking this thinking to extremes, Chordia et al. (2020) study over two million trading strategies from random combinations of accounting variables and basic market variables to test for the presence and magnitude of data mining. Using stricter tests, only 17 (0.04%) of 2.1 million strategies survive. In developing recommendations to underpin a rigorous testing protocol, Harvey et al. (2016) suggest applying higher test statistic thresholds for testing new factors, perhaps using Bayesian adjusted *p*-values to guard against *p*-hacking. Critical *t*-statistic thresholds would increase to 3.0 or even higher under such an approach. Recently, Bryzgalova et al. (2020) use a Bayesian framework to analyze 2.25 quadrillion models and conclude that only 3 factors (‘HML’ value, adjusted size, and adjusted market) appear to be robust.

Focusing on the independent contributions of new factors that are often correlated with “old” factors, Green et al. (2017) suggest that the returns of new candidate factors may need to be orthogonalized against the returns of some but not all pre-existing predictors in order to establish the contribution of new return-predicting signals. In contrast, Bartram and Grinblatt (2018, 2021) bias against data mining by taking an agnostic approach, where modeling choices are non-discretionary and only based on data availability and statistical criteria, biasing against finding predictability.

Overall, recent literature emphasizes the importance of both rigor of statistical methodology combined with solid economic theoretical support in evaluating the performance and contribution of candidate return-predictive factors. The theoretical origins of many factors, including many surviving strategies, are still not well enough understood. A combination of better theoretical support and statistically rigorous backtesting will help researchers interested in factor investing mitigate some of the skepticism that they increasingly encounter from both the academic “gatekeepers” and investment professionals.

## Reality checks for factor investing

Practitioners and institutional investors also need to consider the implementation and feasibility of pursuing factor-based investment strategies. Implementation costs, which include direct costs (such as manager fees), indirect costs (including trading costs), and investment constraints (such as leverage constraints, turnover, and limited capacity) can curtail investors’ appetite for adopting factor-based allocation schemes. Concerns about capacity, measuring how much can be invested in a factor before the additional inflows lead to price pressure and a decline in realized returns, have increased with the popularity of factor investing. But many of these real-world concerns are often neglected in academic work because academics lack the relevant data, and also because investors face different constraints and costs, rendering implementation cost assumptions somewhat subjective. We also discuss integrating the notion of sustainable investing in factor-based investment strategies.

### Measuring factors in practice

While the academic literature has proposed specific measures of an asset’s exposure to returns factors, in practice one would often see a combination of such metrics. For instance, quality factors such as profitability and investment are often backed by several balance sheet indicators, including leverage, earnings quality, return on equity, accruals, asset turnover ratio, and even ESG (Environment, Social, and Governance) based measures that could potentially reflect the safety of a long investment and its susceptibility to large negative returns outcomes. Hsu et al. (2019) express concerns about the definition of quality, commenting that different index providers (e.g., MSCI, FTSE Russell, S&P, Research Affiliates, EDHEC, and Deutsche Bank) have their own combination of signals for measuring the quality premium.

When examining the seven most prominent attributes used by index providers for constructing the quality factor, Hsu et al. (2019) find profitability, accounting quality, payout/dilution, and investment to be more reliable sources of the quality premium than capital structure, earnings stability, and growth in profitability. They emphasize the importance of thorough analysis of each potential signal of a company’s “quality”, the robustness across international markets, and the ability of traditional and non-traditional metrics in capturing the underlying concept being estimated. Thinking of each signal as a noisy indicator of the underlying unobservable construct, composite factors can be thought of as a diversified combination of signals, the performance of which will be enhanced if signals are individually informative and combined in ways that eliminate the overall noise in the measure as much as possible.

Similar diversification considerations apply in measuring value factors. For instance, the S&P 500 Enhanced Value Index combines three fundamental measures, book-to-price, earnings-to-price and sales-to-price. Yet, in the context of the apparently disappearing equity value premium, a recent debate has discussed whether such traditional ratios are still adequate to assess relative company valuation. Arnott et al. (2021) highlight the growing importance of off-balance sheet intangibles, especially in the age of technology-dominated firms. The book equity component of the traditional price-to-book ratio does not capture most internally generated intangible assets, and this has implications for misclassification of growth and value stocks. Therefore, Park (2019) suggests incorporating intangibles into standard value metrics by calculating the intangible-adjusted book-to-market ratio (ibook-to-market). This measure not only outperforms the traditional book-to-market ratio, but also survives in periods when value was reported to be “dead” as a return predictor. While this offers hope for the continued existence of a value premium, it also stresses the need for revisiting traditional factor definitions as business models, accounting recognition, and measurement rules change over time.

### Environmental, social, and governance investing

Sustainable investing aims to create a positive impact on the environment (E), society (S) and corporate governance (G). Unlike traditional style factors, ESG investing focuses on non-financial characteristics, e.g. climate change, waste management, energy efficiency, human capital, labor management, corporate governance, gender diversity, privacy, or data security. While environmental factors such as climate change and waste management attempt to encompass the financial and firm-level consequences of global warming and greenhouse gas emissions, social factors such as human capital and labor management measure a company’s adherence to good workplace practices. With some of the world’s largest asset owners such as the Government Pension Investment Fund of Japan, Norway’s Government Pension Fund Global, and the Dutch pension fund ABP investing trillions of dollars in sustainable investing, it is important to understand the ways in which investors can integrate ESG objectives in their investment mandate without negatively impacting the risk-adjusted returns of their portfolio.

A survey by Krueger et al. (2020) documents that institutional investors believe climate risk to play an important role in the future performance of the firms in their portfolio. Surveying about 413 senior investment professionals who represented about 43% percent of the global institutional assets under management, Amel‐Zadeh and Serafeim (2018) report that institutional investors consider ESG ratings as a proxy for management quality and strongly believe that the rating reflects a firm’s reputational, legal, and regulatory risk. They also report that institutional investors perceive stocks with good ESG ratings to be underpriced, and hence such stocks may offer higher returns than stocks with poor ESG ratings.

However, given the challenges involved in quantifying ESG-related information from firm disclosures, recent academic research has explored the complexities in defining and measuring ESG factors and formulating profitable strategies from the same. In their survey article, Liang and Renneboog (2020) trace the relationship between sustainable investing and firm performance and discuss the caveats of relying on the ratings of ESG rating agencies to compute ESG factor scores. The low correlation between the ESG ratings of different providers underscores the inconsistencies in such ratings. Such a divergence in ESG ratings across vendors and lack of established metrics to measure each of the sustainability topics are some of the reasons why ESG factor research is still inconclusive. Still, from a practitioner perspective, one needs to learn about the return effects of ESG objectives and be in a position to integrate such objectives in portfolio construction without harming risk-adjusted returns.

### Real-world frictions in implementing factors

#### The role of illiquid small-cap stocks

Factor returns in academic studies have often been assessed using equal-weighted portfolios rather than value-weighted portfolios, causing small-capitalization stocks to play a more significant role. This can be important if factor returns depend on firm size, and because implementation costs are generally higher for small companies where stock market liquidity is lower. To assess the importance of equal- versus value-weighting, Hou et al. (2020b) replicate 452 cross-sectional returns results predictors that have been documented in the US equity market. Overall, 64%–85% of these anomalies become statistically insignificant when using more realistic value-weighted portfolios and accounting for liquidity, market microstructure effects, and other trading frictions. The authors document an over-representation of micro-cap stocks and point out how the majority of the evidence from factor studies can be attributed to microcap stocks and hence are unlikely to be exploitable by institutional investors. In related research, Green et al. (2017) study 94 firm characteristics to identify meaningful factors in the cross-section of US stock returns and find that only 25% of them are statistically significant and even fewer after excluding microcap stocks. Several other empirical studies have also noted that the exclusion of microcap stocks leads to similar conclusions, thereby raising questions about the translation of paper profits into real institutional investment gains.

#### Transaction costs

The increased adoption of factor strategies has necessitated the need for real-world cost considerations, increasing the hurdles for factor adoption by practitioners. For instance, some might argue that the high gross returns of a given factor are simply reflective of the transaction cost necessary to arbitrage the very effect. Indeed, building on a mispricing measure (estimated as the deviation of firms’ market values from intrinsic values), Bartram and Grinblatt (2021) study the relation between mispricing and transaction costs in 36 countries and find that gross alphas are positively related to transaction costs. Thus, environments with higher transactions costs also see higher risk-adjusted profits from mispricing. In this sense, limits to arbitrage could explain the Bartram and Grinblatt (2021) mispricing factor and potentially many other anomalies. However, Bartram and Grinblatt (2021) also show that trade implementation is important, and that in some parts of the world, notably Asia Pacific and emerging markets, risk-adjusted profits for their mispricing measure remain significant even after accounting for institutional investors’ estimated trading costs.

To explore whether payoffs to return predictors survive implementation costs, some researchers use market wide data, such as NYSE Trade and Quote (TAQ) to estimate transaction costs, while others use proprietary trading data to assess performance net of trading costs. For instance, Lesmond et al. (2004) use TAQ data to estimate trading costs. They find that the strategies that generate the highest momentum returns are also those with the highest trading costs and conclude that momentum trading is not profitable net of costs. A related perspective is provided by Korajczyk and Sadka (2004) who estimate that the price impact of a fund with over $5 billion under management implies trading costs that exceed the abnormal returns of momentum strategies.

Novy-Marx and Velikov (2016) use Hasbrouck’s (2009) bid-ask spread measure to estimate transaction costs for 23 return predictive factors. They report average trading costs for size, value, and momentum strategies, respectively, of 6 bp, 5 bp, and 48 bp per month, suggesting that size, value, and momentum strategies survive transaction costs. The value-weighted annual returns of value and size strategies drop by roughly 60 bp (from 5.64% to 5.04% for value and from 3.96 to 3.36% for size), while the return on momentum drops from 16% to 8%. Frazzini et al. (2018) use live proprietary trading data across international equity markets and find that real-world costs based on live trades are very different than those estimated in the literature from daily or intra-day data. Using this data, Frazzini et al. (2014) show that size, value, and momentum survive transaction costs, unlike the short-term reversal factor, which has high turnover.

Hence, we observe a disconnect between academic work, which tends to conclude that transaction costs erode most the factors’ excess returns, and practitioner work, which argues that academics are perhaps too conservative. This disconnect is perhaps related to many academic studies implicitly assuming a too aggressive trading style that can often lead to crossing the spread. Conversely, a passive execution style seems to curb market impact while retaining most of the factor signals’ predictability. In this context, Frazzini et al. (2018) point out that in their live trade data, trades are often executed as limit orders that remain on the book for some time. In practice, portfolio construction naturally needs to adapt to portfolio size for not trading a significant part of a given stock’s available dollar volume.

Such analyses help identify whether single factor strategies are really profitable on a standalone basis. Identifying the transaction cost drivers has been shown to also positively alter multi-factor-based portfolio allocations by enhancing the diversification potential. DeMiguel et al. (2020a) examine the impact of transaction costs using characteristics-based factor selection with a lasso penalty. Before transaction costs, 6 of 51 characteristics are significant in a multivariate parametric portfolio policy, while 15 are significant after transaction costs. This seemingly counter-intuitive result is due to the increased benefits from trading diversification due to offsetting positions attributable to different stock characteristic-based factors. The authors argue that trades in the underlying stocks required to rebalance different characteristics often cancel each other out, and thus combining a larger number of characteristics allows one to substantially reduce the quantum of transactions, and hence transaction costs. Such findings from the literature may aid in improving the profitability of factor strategies.

#### Capacity constraints

The capacity constraints of many factor-based investing strategies also have important implications for realistic assessments of factor performance. While different definitions of capacity exist in the literature, the capacity of a strategy can be understood as the volume of additional capital that can be invested in a strategy before it becomes unprofitable. Thus, capacity constraints reflect limits on the size of trades intended to increase factor exposure due to market impact effects. This issue is investigated by Novy-Marx and Velikov (2016), who document a negative relation between factor turnover and capacity: low turnover strategies, such as value, size, and profitability, have capacities of about $21 billion, $20 billion, and $131 billion, respectively, while mid-turnover strategies such as idiosyncratic volatility and momentum have capacities of about $1.51 billion and $5 billion, respectively.

Ratcliffe et al. (2017) use proprietary high frequency trading data and find that the capacity of factor strategies varies with the trading horizon. Considering a 1-day trading horizon, the MSCI USA Minimum Volatility index has a break-even capacity of around $1.3 trillion. For a 5-day trading horizon, it jumps to $6.7 trillion. Other factors such as size, value, quality, momentum, and even multi-factor strategies display similar behavior, suggesting that they have very high capacities. However, practitioners criticize this study because of highly concentrated positions associated with higher capacity. An active factor investing strategy that trades multiple times a year seems to be a better alternative (Blitz and Marchesini 2019).

### Factor crowding

Crowded trades have been of concern to institutional investors as such crowding has been shown empirically to hamper factor performance. Factor crowding occurs when any given factor experiences a huge influx of investment. For example, Dimson and Marsh (1999) note the disappearance of the size premium after a surge in the popularity of small cap funds. Momentum has been similarly criticized for being vulnerable to poor performance after gaining popularity. Researchers have supported this view by identifying that momentum’s performance is strongly affected by crowding (Lou and Polk 2014). Concerns about capacity in factor investing have been exacerbated in recent years as a result in the growth of investment in exchange-traded funds tracking style factor indices. Crowded factors not only perform poorly but may also experience increased volatility and drawdowns.

As crowded trades continue to pose a concern for practitioners, the academic literature has identified different ways of measuring crowdedness in factors. Since the ideology of factors stems from exploiting common patterns, a reliable measure of crowdedness could be identifying the number of investors chasing (or wanting to exit) a particular strategy. Given significant commonality of factors in their alpha models, institutional investors are likely to hold similar stocks and be affected by crowded trades, e.g. in case of fire sales (Jotikasthira et al. 2012). Common ownership of international stocks is a predictor of returns that can be quantified in an institutional ownership return measure (Bartram et al. 2015).

Interestingly, different asset classes are impacted differently by factor crowding. Baltas (2019) studies the impact of market crowding on equity momentum, equity low beta, equity quality, FX momentum, and commodity momentum. In line with the views of Stein (2009), Baltas (2019) finds that FX momentum and commodity momentum face lower drawdowns than equity momentum over six-month to one-year investment horizons when strategies are crowded. However, in the subsequent year, equity momentum strategies begin to outperform, indicating underperformance is short-lived. Relatedly, DeMiguel et al. (2020b) show that crowding concerns can be alleviated by trading diversification and other institutions exploiting strategies that, when implemented concurrently, reduce their price impact. Overall, the available evidence suggests that factor crowding may not be a valid reason for long horizon investors to avoid factor investing.

### Short-selling

The long-short investment strategies commonly investigated in academic return-predictive factor studies inherently assume that both the long leg and the short leg contain relevant factor-related information. Hence, investors can enjoy factor-related returns relative to a cash benchmark through zero investment long-short hedge portfolios; or relative to a benchmark index by overweighting (underweighting) stocks in the long (short) leg relative to benchmark weights. This methodology has been subject to criticism as long and short portfolios may be subject to different return dynamics. To illustrate, the long and short portfolios of value, size, and momentum strategies exhibit differential exposures to term structure risk (Aretz et al. 2010). This leads to an asymmetric behavior of the long and short side, which has been repeatedly observed in the academic literature.

While the contribution of the short leg to the performance of long-short strategies is evident from several academic findings, investors might be limited in their ability to short stocks due to short selling constraints, borrowing costs, risks of short-selling in the form of short squeezes, etc. These limits to arbitrage can prevent sophisticated investors from trading profitably against anomalies (Miller 1977). Indeed, Stambaugh et al. (2012) show that Fama–French 3-factor alphas are larger for the short leg than the long leg of the investment strategy for all but one of 11 anomalies. They further show that the short leg returns are lower when market sentiment is high. In fact, they suggest that short-selling could even enhance factor performance when combined with market sentiment and note an increase in anomaly returns especially during high-sentiment periods as the extant mispricing in those anomalies would be higher, translating into higher returns. Similarly, Chu et al. (2020) document the causal effect of short-selling constraints on asset pricing anomalies, with the introduction of short-selling constraints shown to affect only the short legs of anomaly portfolios, significantly reducing risk-adjusted long-short portfolio performance.

Shorting stocks also entails borrowing costs and risk associated with the liquidity of stocks in the short leg (Diether et al. 2008). Kim and Lee (2019) report shorting costs to be 0.10% per month, which is about 40% of gross long-short returns of 14 factors such as return on equity, return on assets, momentum, etc. Limitations to the ability to liquidate short positions or “short squeezes” expose investors to unintended sources of risk, which can be circumvented by underweighting the stocks in the portfolio. Such findings are often overlooked and are an important limitation in translating many academic findings into practice (Patton and Weller 2020).

### Are there benefits to factor timing?

Time variation in factor performance presents a major challenge to institutional investors, since factors can experience extended periods of underperformance. While academics have the luxury of being able to look at long-term averages, the need to document favorable performance to clients over relatively short reporting intervals creates risk for real-world asset managers. Consequently, it is conceptually appealing to avoid such painful episodes of underperformance by actively timing factor weights in an investment portfolio, moving into a factor when it is likely to perform well and out when it is expected to underperform.

Recent factor timing research focuses on utilizing past factor performance to predict future factor performance. To illustrate, Avramov et al. (2017) show that short-term factor momentum strategies outperform an equal-weight factor allocation in a universe of 15 well-known factor strategies. In a similar vein, Gupta and Kelly (2019) offer international factor momentum evidence in a comprehensive set of 65 characteristics-based factors around the globe, where factor momentum is found to add significantly to investment strategies based on traditional momentum, industry momentum, value, and other commonly studied factors. Similarly, Arnott et al. (2019) find that factor momentum is pervasive across all factors and that factor momentum fully subsumes industry momentum. Ehsani and Linnainmaa (2019) explain how momentum in factors translates to momentum in individual stocks and argue that factor momentum fully explains individual stock momentum.

Expected factor performance, and hence factor weights, are related to general economic and market conditions—for example momentum and value tend to perform better in bull markets, while quality and minimum volatility perform better in bear markets. As a case in point, factor timing became very popular during the global financial crisis, where markets were more driven by policy and macroeconomic events rather than firm fundamentals. Interest-rate regimes (Muijsson et al. 2014) and business cycles (Grant et al. 2012) have also been shown to affect the performance of different factors. Grant et al. (2012) document strong predictability for carry and momentum strategies with the business cycle (using dividend yield, short rate, term spread, and default spread as instruments) and liquidity indicators have predictive power for factor returns across a range of asset classes.

While some of this evidence suggests that factor timing is possible if the economic and market determinants of factor performance can be anticipated in advance, the evidence is mixed on the possibility of timing factors profitably in practice. In this regard, Dichtl et al. (2019) explore the value-added of active factor allocation strategies for investible global equity factors. They find equity factors to be related to lagged fundamental and technical time-series indicators and to characteristics such as factor momentum and crowding. Yet, such predictability is difficult to exploit after transaction costs. The consensus among many practitioners is that the higher turnover and associated costs of dynamic factor allocation strategies outweighs the gross return benefits that can be expected (Asness et al. 2017). Supporting the findings of Van Gelderen and Huij (2014), Van Gelderen et al. (2019) argue that investors are better off by choosing a buy-and-hold strategy compared with dynamic factor allocation strategies. Unfortunately for investors, factor timing seems “clear in hindsight but hazy ahead” (Vanguard Investment Strategy Group 2019).

## Conclusion

The overall findings from academic factor studies can be summarized as follows: the empirical asset pricing literature has spawned a multitude of factors to explain the cross-section of returns, and the past three decades have witnessed a heightened proliferation of new factors (Cochrane 2011). However, only a small number of dominant factors survive after careful and rigorous testing of significance, controlling for data snooping and other research design biases, considering real-world constraints, and careful examination of the incremental contribution of specific factors to return predictability. Identifying such “true” factors is challenging, especially in light of data-mining concerns.

Recent meta-studies have proposed new ways of dealing with these issues. Most are fueled by data-driven and computationally intensive methods that would deem many of the factors insignificant. In the emerging age of alternative (and potentially big) data, such data mining concerns are likely to be exacerbated as researchers constantly conceive new factors, e.g., by applying ML techniques to big data and NLP techniques to unstructured text data or invoking the various facets of available ESG criteria. At the same time, improved understanding of the risk-return relationship helps in uncovering the underlying common factors, the associated premia, and the competing explanations for their existence. Factors that capture these premia are expected to aid institutional investors in structuring portfolio allocations.

Although not immune to episodes of poor performance, factor investing has survived turbulent times such as the global financial crisis and the ongoing Covid crisis. Part of this relative success is due to factors’ underlying building blocks not moving in lockstep and thus offering diversification and downside protection benefits. Whereas, the longer-term performance of factor strategies arises from the reward associated with bearing risk, exploiting structural impediments, or behavioral biases of investors. A factor-based approach can cater to institutional investors’ specific risk-return objectives at improved transparency. Indeed, multi-asset multi-factor-based investment approaches can help maximize portfolio diversification relative to traditional asset allocation by combining asset class and style factors.

From an institutional investor perspective, many factors discovered in the academic literature may not be exploitable under real-world conditions. Taking into account such challenges in translating paper profits to reality, this literature points to the importance of parsimonious and implementable asset pricing models. Rigorous testing procedures have found that the premia of spurious factors vanish for value-weighted portfolios or after excluding small-cap stocks. Any remaining profits are often accounted for by limits to arbitrage and related transaction costs. Apart from rare attempts to measure mispricing, distinguishing between risk and mispricing remains a key challenge, with some recent work suggesting that common anomalies capture time-varying factor risk. It is still of huge concern which factors are likely to pass all real-world tests, especially to investors, who are often bewildered by all the different possible options.

## Appendix

### Equity style factor definitions

- The *Equity Value* factor is based on the MSCI value metric, which uses three characteristics: book value-to-price, 12-month forward earnings-to-price, and dividend yield. Combining these three metrics, MSCI computes each securities’ value and growth score and allocates the security into a value or a growth index. Security weights are computed by sorting securities according to their distance from the origin and other investment constraints, see MSCI Index methodology documents^1^ for more specific details. The index is reviewed and rebalanced semi-annually in order to reflect changes in the underlying markets, creating only limited index turnover.
- The *Equity Quality* factor is based on the MSCI quality metric, which combines information from three fundamental variables: high return on equity (ROE), stable year-over-year earnings growth, and low financial leverage. Offering high trading liquidity and investment capacity, the quality index is constructed using a composite Z-score computed by averaging the Z-scores of these three variables. The weights of individual securities are simply the product of this quality Z-score and market capitalization weight of the security in the parent index. Capping issuer weights at 5%, the index has moderate index turnover and is rebalanced semi-annually.
- The *Equity Momentum* factor is based on the MSCI momentum index and uses the securities’ recent 12-month and 6-month risk-adjusted price performance to compute a momentum score. MSCI computes the security weights by multiplying the momentum score with the market capitalization weight in the parent index.
- The *Equity Low-Volatility* factor is based on the MSCI low volatility index, which aims to minimize the return variance for a given covariance matrix of returns and hence takes a different approach with respect to other style factors. The index targets lower beta and volatility than its parent index and uses the Barra optimizer to optimize its parent index for the lowest absolute volatility with a certain set of constraints.

## Non-equity style factor definitions

All non-equity style factors and their definitions were sourced from Goldman Sachs except for Commodity Quality.

### Rates factors

- The *Rates Momentum* factor capitalizes on the persistence of trends in short- and long-term interest rate movements. On a daily basis, the strategy evaluates the recent performance of a number of futures contracts for the US, Germany, Japan, and the UK. It then takes either a long or short position on each, depending on whether actual performance has been positive or negative.
- The *Rates Quality* factor capitalizes on the observation that risk-adjusted returns at the short end of the curve tend to be higher than those at the long end. A leveraged long position on the former versus the latter tends to capture positive excess returns as compensation for the risk premium that stems from investors having leverage constraints and favoring long-term rates. The interest rates curve strategy enters a long position on five-year US bond futures, and a short position on 30-year bond futures, as well as a long position on five-year German bond futures and a short position on ten-year German bond futures, rolling every quarter. The exposure to each future is adjusted to approximate a duration-neutral position.
- The *Rates Value* strategy attempts to capture the bond risk premium as compensation beyond the expected rates path. The risk premium of Rates Value is defined as the difference between bond yield (using ten-year futures) and inflation expectations (provided by Consensus Economics) for a number of G8 government bonds. The risk management of the strategy involves the construction of a portfolio on a daily basis to maximize the exposure to the risk premium, subject to constraints on risk, leverage, and potentially beta to a benchmark. The execution is smoothed over 22 days with a view of limiting turnover and transaction costs.
- The *Rates Carry* strategy benefits from upward-sloping yield curves as compensation for bearing duration, inflation, and illiquidity risk. Higher carry tends to be compensation for being long riskier assets. The risk premium of Rates Carry is defined as the difference between bond yield (using 10-year futures) and funding cost plus the roll down of a number of G8 government bonds. The risk management of the strategy involves the construction of a portfolio on a daily basis to maximize the exposure to the risk premium, subject to constraints on risk, leverage and potentially beta to a benchmark. The execution is smoothed over 22 days with a view of limiting turnover and transaction costs.

### FX factors

- The *FX Carry* strategy benefits from the overestimation of the actual depreciation of future FX spot by the FX forwards of high-yielding currencies. Ranked using the implied carry rate (FX forwards versus FX spot) of a number of currencies (G10 and EM) against the USD, the strategy goes long on single currency indices (which roll FX forwards) for the currencies with the highest carry, and short on single-currency indices for the currencies with the lowest carry. It is reviewed and rebalanced on a monthly basis.
- Relying on the mean reversal of exchange rates, the *FX Value* strategy ranks the currencies according to the valuation measure (based on GS DEER, Dynamic Equilibrium Exchange Rate model) of a number of currencies (G10 and EM) against the USD. Rebalanced monthly, the strategy goes long on single-currency indices (which roll FX forwards) on the most undervalued currencies, and short on single-currency indices on the most overvalued currencies.
- Capitalizing on the persistence of trends in forward exchange rate movements that are driven by both carry and spot movements, the *FX Momentum* strategy evaluates the recent performance of 27 currencies against the USD and is rebalanced on a daily basis. Long/Short positions are determined based on whether actual performance has been positive or negative.

### Commodity factors

- *Commodity Carry* captures the tendency of commodities with tighter time spreads to outperform due to low inventories that drive both back-dated futures curves and price appreciation, and to buy demand from consumer hedgers for protection against price spikes in undersupplied commodities. The strategy goes long on the top third and short on the bottom third of the 24 commodities from the S&P GSCI universe, ranked by annualized strength of front month time spreads. The strategy is rebalanced daily based on signals over the last ten days. The strategy is net of cost.
- *Commodity Value* uses the weekly Commodity Futures Trading Commission (CFTC) positioning data to determine long and short positions. It will take long positions in commodities where the speculative positions are the most short, and short positions in commodities where the speculative positions are the most long.
- *Commodity Momentum* in commodity returns reflects initial underreactions, or subsequent overreactions, to changes in demand. Increases or decreases in supply can take many years to implement and may subsequently overshoot the required changes to match demand. The strategy goes long on the top third and short on the bottom third of the twenty-four commodities from the S&P GSCI universe, ranked by rolling one-year excess returns of each commodity. The strategy is rebalanced daily based on signals over the last ten days. The strategy is net of cost.
- *Commodity Quality* is long the Bloomberg Roll Select Commodity Index and short the Bloomberg Commodity Index. The Bloomberg Roll Select Commodity Index is a of the Bloomberg Commodity Index (BCOM) that aims to mitigate the effects of contango on index performance. To do this, the index rolls into the futures contracts for each commodity with the most backwardation or least contango. The contract selection process is performed on the fourth business day of each month.
  **Table 3:** Style factor indices used for Fig. 3
  Style factor	Equity	Fixed income	Commodity	FX
  Carry		GS interest rate carry 03	GS macro carry index RP14	GS FX carry C0115
  Value	MSCI ACWI value	GS interest rate value 03	GS commodity COT strategy COT3	GS FX value C0114
  Momentum	MSCI ACWI momentum	GS interest rate trend 03	GS macro momentum index RP15	GS FX Trend C0038
  Quality	MSCI ACWI quality	GS interest rate curve C0210	Bloomberg roll select commodity Index minus Bloomberg Commodity Index	
  Low Volatility	MSCI ACWI Minimum volatility			
  The table provides details on the style factor series used for computing the metrics shown in Fig. 3. Equity style factors and Commodity Quality are sourced from Bloomberg and all other non-equity style factors are sourced from Goldman Sachs (GS)
